# Determinants of the uptake of intermittent preventive treatment of malaria in pregnancy with sulphadoxine pyrimethamine in Sabatia Sub County, Western Kenya

**DOI:** 10.1186/s40249-021-00887-4

**Published:** 2021-08-06

**Authors:** Joshua A. Mutanyi, Daniel O. Onguru, Sidney O. Ogolla, Lawrence B. Adipo

**Affiliations:** 1 Department of Public and Community Health, School of Health Sciences, Jaramogi Oginga Odinga University of Science and Technology, P.O. Box 210, Bondo, 40601 Kenya; 2 Department of Public and Community Health, School of Health Sciences, Jaramogi Oginga Odinga University of Science and Technology, P.O. Box 210, Bondo, 40601 Kenya; 3 Kenya Medical Research Institute, Centre for Global Health Research, P.O. Box 20778, Kisumu, 00202 Kenya

**Keywords:** Malaria, Pregnancy, Intermittent preventive treatment, Sulphadoxine pyrimethamine, Sabatia

## Abstract

**Background:**

Annually, 125.2 million pregnant women worldwide risk contracting malaria, including 30.3 million and 1.5 million in Sub-Saharan Africa and Kenya respectively. At least three doses of sulphadoxine pyrimethamine for intermittent preventive treatment of malaria in pregnancy (IPTp-SP) is recommended for optimal benefit. Kenya recorded low IPTp-SP optimal uptake in 2015. This study investigated the prevalence of and factors influencing IPTp-SP optimal uptake in Sabatia Sub County, Western Kenya.

**Methods:**

A cross-sectional study was conducted in Sabatia Sub County from April to October 2020. Using a validated semi-structured questionnaire, data were obtained from 372 randomly sampled post-delivery women aged 15–49 years with live birth within one year preceding the study. Women on cotrimoxazole prophylaxis during pregnancy were excluded. Pearson Chi-square and Fisher’s Exact test were measures of association used. Binary logistic regression analysed predictors of optimal IPTp-SP uptake.

**Results:**

Optimal IPTp-SP uptake was 79.6%, 95% *CI* 75.5%–83.7%. Predictors of IPTp-SP optimization were gestational age at first antenatal care (ANC) visit (*P* = 0.04), frequency of ANC visits (*P* < 0.001), maternal knowledge of IPTp-SP benefits (*P* < 0.001), maternal knowledge of optimal sulphadoxine pyrimethamine (SP) dose (*P* = 0.03) and SP administration at ANC clinic (*P* = 0.03). Late ANC initiators were less likely to receive optimal IPTp-SP (a*OR* = 0.4, 95% *CI* 0.2–0.9). Odds of optimizing IPTp-SP increased among women with ≥ 4 ANC visits (a*OR* = 16.7, 95% *CI* 7.9–35.3), good knowledge of IPTp-SP benefits (a*OR* = 2.4, 95% *CI* 1.3–4.5) and good knowledge of optimal SP dose (a*OR* = 1.9, 95% *CI* 1.1–3.4). Women who never missed being administered SP were highly likely to receive optimal IPTp-SP (a*OR* = 2.9, 95% *CI* 1.1–7.2)

**Conclusions:**

This study has found high IPTp-SP optimal uptake in the study area. Efforts should be directed towards early and more frequent ANC visits. Intensive and targeted health education is required. It’s fundamental to adequately stock and consistently administer SP. Future studies considering larger samples and health workers’ perspectives of the health system delivery factors are recommended.

**Graphical abstract:**

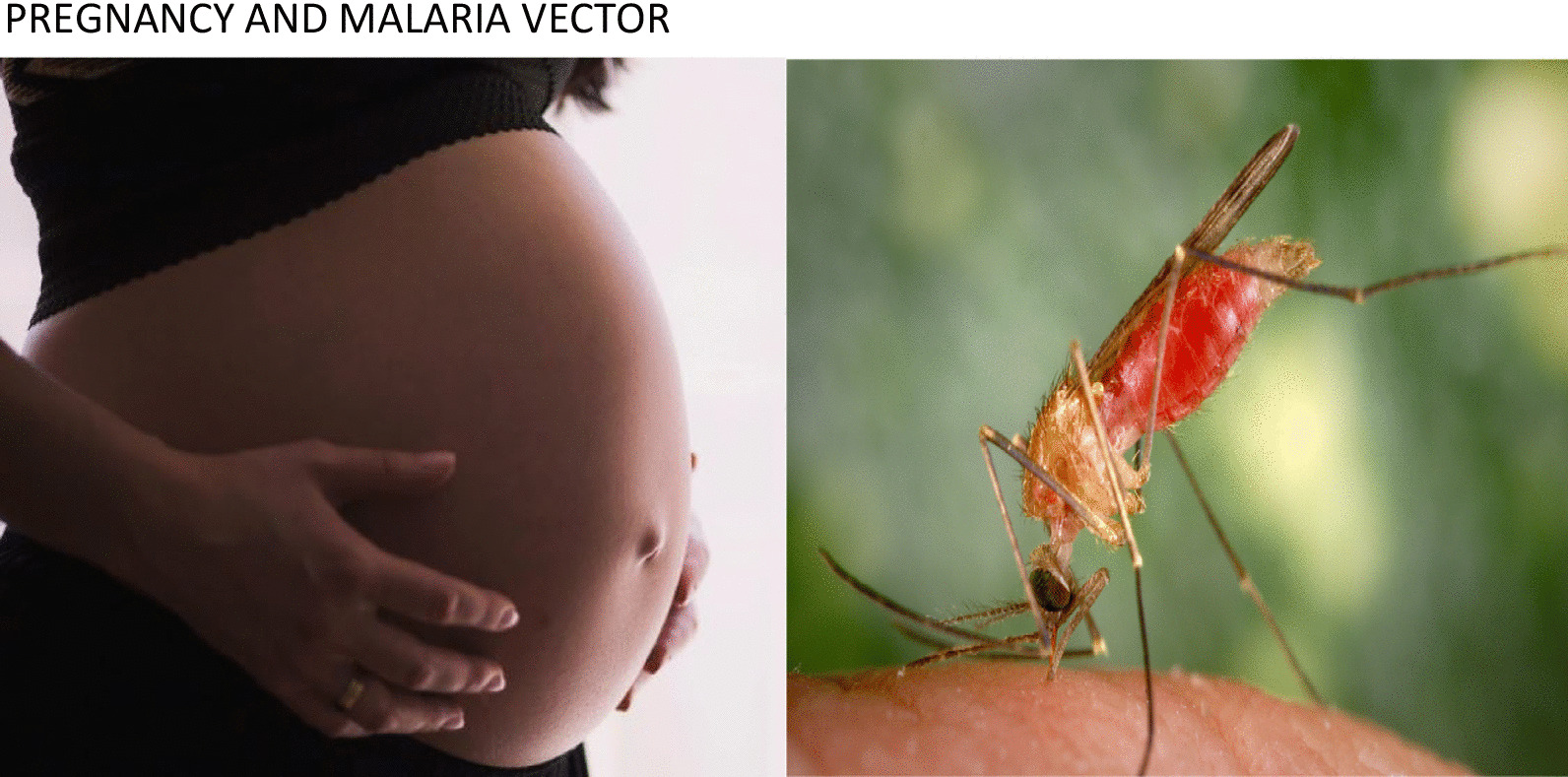

**Supplementary Information:**

The online version contains supplementary material available at 10.1186/s40249-021-00887-4.

## Background

Malaria in pregnancy (MiP) remains a major public health problem. It is of considerable importance particularly when preventive interventions are inadequate [1]. In malaria endemic areas, MiP risk is 50% higher among pregnant women [2] due to pregnancy induced lowered immunity [3]. MiP causes anaemia, which in turn raises maternal and neonatal mortality risk [2]. Other negative consequences of MiP include haemorrhage, intrauterine growth retardation, still birth, preterm delivery, placental malaria, miscarriage, low birth weight (LBW) and congenital infections [2, 4].

Globally, 125.2 million pregnant women risk contracting malaria yearly, including 30.3 million in stable malaria transmission zones of Sub-Saharan Africa (SSA) [5]. Approximately 40% of all pregnancies in SSA would experience *Plasmodium falciparum* placental infection in the absence of MiP preventive interventions causing 900 000 LBW deliveries annually [6]. In Africa, about 10 000 women and 75 000 to 200 000 infants die yearly due to MiP while LBW from *P. falciparum* infections during pregnancy causes nearly 100 000 neonatal deaths [2]. In Kenya, about 1.5 million pregnancies occur yearly, with 44% in moderate to intense malaria endemic areas [3]. The country’s MiP prevalence was 4918 cases per 100 000 persons in 2018 [7]. Intermittent preventive treatment of malaria in pregnancy (IPTp) with sulphadoxine pyrimethamine (SP) is recommended by World Health Organization (WHO) for controlling MiP [8].

The WHO recommends provision of IPTp-SP to all pregnant women in moderate to high malaria transmission areas starting earliest in the second trimester at each scheduled antenatal care (ANC) visit until delivery, observing at least one month interval between the SP doses [8]. Kenya’s Ministry of Health recommends at least three IPTp-SP doses (IPTp-SP3+) to pregnant women in malaria endemic areas [9]. The drug is safe and can be taken with or without food. However, WHO does not recommend SP to Human Immunodeficiency Virus (HIV) infected pregnant women on cotrimoxazole prophylaxis due to the drugs’ redundant mechanisms of action and synergistic worsening of adverse drug reaction [8]. SP remains cost effective and efficacious for IPTp [10, 11]. IPTp-SP has been linked to reduced MiP cases, lower placental malaria risk and reduced neonatal deaths [12]. The intervention was associated with increases in maternal haemoglobin and birth weight even in areas that experienced SP resistance in SSA [13].

Optimal IPTp-SP uptake remains below global targets in SSA [14]. Two different studies reported IPTp-SP3+ uptake levels of 22% and 29.5% in Africa [15, 16]. In Malawi and Uganda, the IPTp-SP optimal uptakes were 29.8% and 18% respectively [17, 18]. Other studies in Ghana and Sierra Leone reported high optimal IPTp-SP uptake levels of 71%, 76.4% and 93.24% [16, 19, 20]. In Kenya, the 2015 national survey found IPTp-SP3+ uptake to be 37.5% [21], far below the 80% national target [9]. However, IPTp-SP3+ uptake in specific areas in the country remained unknown and studies on the same after malaria policy revision in 2014 have been limited.

Socio demographic and obstetric characteristics have been found to influence the uptake of IPTp-SP by pregnant women [16–18, 22]. Knowledge related factors such as maternal knowledge of MiP and IPTp-SP have also been demonstrated to predict IPTp-SP optimal uptake [23, 24]. In SSA, poor IPTp-SP coverage levels and many missed opportunities for IPTp-SP delivery have been attributed to health care system inadequacies [25–27]. Despite this, determinants of IPTp-SP3+ uptake in Sabatia Sub County were unclear and unknown. This study sought to investigate the prevalence of and factors influencing IPTp-SP3+ uptake in the Sub County. Understanding the prevalence and determinants of IPTp-SP3+ uptake is core for making decisions and setting priorities towards improved access to and optimization of the intervention.

## Methods

### Study site and study design

The study site was Sabatia Sub County, Vihiga County in Western Kenya. It is a malaria endemic zone. In 2018, its estimated pregnant women’s population was 5117 [7]. It’s mainly rural and most residents are in agriculture and rural development sector. A household-based cross-sectional study using a quantitative approach was conducted from April to October 2020. Cross-sectional study design was used due to its relevance in estimating prevalence and measuring both outcome and exposure variables simultaneously.

### Target and study population

The target population was pregnant women aged 15–49 years. However, the study population included post-delivery women aged 15–49 years with live birth within one year preceding the study, were residents of Sabatia Sub County during their last pregnancy for ≥ 9 months before the study and consented to participate. The study measured IPTp-SP uptake at pregnancy end, hence the use of post-delivery women. HIV-infected women on cotrimoxazole prophylaxis during their last pregnancy and those with mental disorders were excluded.

### Study variables

The dependent variable was number of IPTp-SP doses received. Though a numerical variable, a dichotomy was created to have sub-optimal IPTp-SP uptake (≤ 2 doses) and optimal IPTp-SP uptake (≥ 3 doses). Table 1 shows independent variables and their definitions.

**Table 1:** Definition of independent variables

Variable	Definition
Sociodemographic	
Maternal age	Age in years, further grouped 15–24, 25–34 and 35+ years
Educational level	Highest level attained, classified as none, primary, secondary and tertiary
Marital status	Either married or unmarried
Residence	Either rural or urban
Employment status	Categorized as unemployed, informal employment or formal employment
Religion	Grouped as none, Roman Catholic, Protestant/other Christian or Muslim
Obstetric	
Parity	Number of live births, further categorized as 1, 2 and 3 +
Gestational age at 1st ANC visit	Pregnancy age at 1st ANC visit, further grouped as ≤ 16 or > 16 weeks
Frequency of ANC visit	Total number of ANC visits, further classified as < 4 and ≥ 4 weeks
Experience of SP side effects	Whether a woman experienced SP side effects or not
Knowledge related	
Knowledge of MiP dangers	≥ 3 correct responses indicated adequate knowledge, 2 correct responses indicated moderate knowledge, 1 correct response indicated low knowledge, otherwise unknowledgeable
Knowledge of MiP prevention	≥ 3 correct responses indicated adequate knowledge, 2 correct responses indicated moderate knowledge, 1 correct response indicated low knowledge, otherwise unknowledgeable
Knowledge of IPTp-SP benefits	Identifying malaria prevention as the benefit of IPTp-SP indicated good knowledge, otherwise poor knowledge
Knowledge of IPTp-SP start	Mentioning gestation age between 13 to 16 weeks as the best time to start IPTp-SP indicated good knowledge, otherwise poor knowledge
Knowledge of optimal SP dose	Mentioning uptake of SP doses in the range of 3 to 8 during the entire pregnancy period indicated good knowledge, otherwise poor knowledge
Health service delivery	
SP administration at ANC clinic	Either a woman ever missed or never missed being issued SP
ANC clinic working hours	Whether ANC services were accessible half day or whole day
Maternal services fee	Whether a woman ever paid for ANC services or not
ANC clinic waiting time	Time in minutes spent queueing before being attended to at ANC clinic, further grouped as ≤ 30 and > 30 min
Water provision at ANC clinic	Whether ANC clinic provided water for taking SP always, sometimes or never, as reported by a pregnant woman
Clean water at ANC clinic	Whether water for taking SP at ANC clinic was clean always, sometimes or never, as reported by a pregnant woman
Clean cups at ANC clinic	Whether cups/glasses for taking SP at ANC clinic were clean always, sometimes or never, as reported by a pregnant woman
Enough cups at ANC clinic	Whether cups/glasses for taking SP at ANC clinic were enough or not, as reported by a pregnant woman
Health worker-client relationship	On most occasions; 1. Whether a health worker greeted a pregnant woman before attending to her, 2. Whether enough time was spent attending to the woman, 3. Whether the woman was counselled/ educated on IPTp-SP benefits, 4. Whether the woman was given a chance to ask questions, 5. Whether the woman felt comfortable asking questions, 6. Whether her questions were answered satisfactorily, 7. Whether the health worker ever shouted at the woman. At least four positive responses to the first six questions and a negative response to the last question indicated good relationship, otherwise poor

### Data collection

Face to face interviews of post-delivery women by trained research assistants were conducted using validated semi-structured questionnaires (Additional file 1). Data were collected on both the dependent and independent variables. ANC clinic attendance booklets were used to verify data on SP doses received, ANC visits made and gestational age at first ANC visit after asking study participants on the same. Each participant’s responses and booklet information were compared to determine their accuracy. All the women studied had the clinic attendance booklets.

### Sample size and sampling

Sample size was calculated using Cochran’s (1977) formula for categorical data. Further, Cochran’s (1977) correction formula was applied since the initial obtained sample size exceeded 5% of the population [28, 29]. The sample size was determined based on optimal IPTp-SP prevalence of 37.5% as per the 2015 national survey [21], a 95% confidence level, a 5% precision and a 10% non-response rate. The final sample size was 372.

Simple random sampling was applied. The process began by identifying, listing and informing all potential participants about the study. After seeking permission from relevant authorities, Community Health Volunteers’ registers of pregnant women were used to develop a sampling frame of 3091. Next, a consecutive identification number from 1 to 3091 was assigned to each recruited participant. Microsoft Excel (Microsoft Corporation, Redmond, Washington, US) version 2016 “RANDBETWEEN()” function was used to randomly sample 372 participants from the sampling frame.

### Statistical analysis of data

Descriptive analyses of continuous and categorical variables were done by calculating means and proportions respectively. Pearson Chi-square test and Fisher’s Exact test where appropriate were used to compare differences in various predictors of interest. Binary logistic regression models were fitted to determine the relationship between IPTp-SP uptake and the predictors. Predictors with *P* < 0.15 from step-wise regression were included in the multivariable logistic regression model. Crude odds ratios (c*OR*) and adjusted odds ratios (a*OR*) were reported and all predictors with *P* < 0.05 were considered to be independently associated with IPTp-SP optimal uptake. Statistical analyses were performed using Stata version 14.0 (Stata Corp., College Station, TX).

### Ethical considerations

The study was approved by Jaramogi Oginga Odinga University of Science and Technology Ethics Review Committee (Approval Number: 7/17/ERC/11/3/20-21) and the National Commission for Science, Technology and Innovation in Kenya (License Number: NACOSTI/P/20/5052). Informed consent was sought from all respondents using an approved consent form. Privacy and confidentiality of the study participants and all raw data were strictly observed. Sabatia Sub County Medical Officer of Health permitted the study (Additional file 1).

## Results

### Characteristics of the respondents

A total of 372 participants were enrolled in the study. Table 2 summarizes their general characteristics.

**Table 2:** General characteristics of participants

Characteristics	*n* (%)
Maternal age (years), mean (± *SD*)	26.3 ± 5.7
Age category	
15–24 years	160 (43.0)
25–34 years	173 (46.5)
≥ 35 years	39 (10.5)
Marital status	
Unmarried	87 (23.4)
Married	285 (76.6)
Woman's education level	
Primary	142 (38.2)
Secondary	178 (47.8)
Tertiary	52 (14.0)
Woman's employment status	
Not employed	252 (67.7)
Informal employment	95 (25.6)
Formal employment	25 (6.7)
Religion	
Catholic	20 (5.4)
Protestant	350 (94.1)
Muslim	2 (0.5)
Ward of residence	
Busali	85 (22.9)
Wodanga	49 (13.2)
North Maragoli	31 (8.3)
Chavakali	64 (17.2)
Sabatia	60 (16.1)
Lyaduywa Izava	83 (22.3)
Residence	
Rural	287 (77.2)
Urban	85 (22.8)
Gestation age at 1st ANC (weeks), mean (*SD*)	16.3 ± 6.0
Frequency of ANC visits, median (IQR)	5 (2)
Parity	
1 child	142 (38.2)
2 children	82 (22.0)
3+ children	148 (39.8)
Experience of SP side effects	
No	197 (53.0)
Yes	173 (46.5)
Not applicable	2 (0.5)
Knowledge of MiP dangers	
Unknowledgeable	112 (30.1)
Low knowledge	136 (36.5)
Moderate knowledge	94 (25.3)
Adequate knowledge	30 (8.1)
Knowledge of MiP prevention	
Unknowledgeable	5 (1.3)
Low knowledge	216 (58.1)
Moderate knowledge	128 (34.4)
Adequate knowledge	23 (6.2)
Knowledge of IPTp-SP benefits	
Poor	77 (20.7)
Good	295 (79.3)
Knowledge of IPTp-SP start	
Poor	318 (85.5)
Good	54 (14.5)
Knowledge of optimal SP dose	
Poor	183 (49.2)
Good	189 (50.8)

ANC: Antenatal care; IPTp-SP: Intermittent preventive treatment of malaria in pregnancy with sulphadoxine pyrimethamine; IQR: Interquartile range; *SD*: Standard Deviation; SP: Sulphadoxine pyrimethamine

The participants’ average age was 26.3 [Standard Deviation (*SD*) = 5.7] years, with majority (173, 46.5%) aged 25–34 years. Most (285, 76.6%) participants were married, majority (178, 47.9%) attained secondary education, about two-thirds (252, 67.7%) were unemployed, the highest (350, 94.1%) were protestants, most (85, 22.9%) lived in Busali ward and over three-quarters (287, 77.2%) were rural residents (Table 2). Mean gestation age at ANC initiation was 16.3 (*SD* = 6.0) weeks and median ANC visitation was 5 [Interquartile Range (*IQR*) = 2] visits. In total majority (148, 39.8%) had a parity of 3+ , over a half (197, 53.0%) never experienced any SP side effect, most (136, 36.6%) had low knowledge on MiP dangers, majority (216, 58.1%) had low knowledge of MiP prevention, over three-quarters (295, 79.3%) had good knowledge of IPTp-SP benefits, most (318, 85.5%) didn’t know the best IPTp-SP start date and half (189, 50.8%) had good knowledge of optimal IPTp-SP dosage (Table 2).

### Prevalence of IPTp-SP optimal uptake

**Fig. 1:**
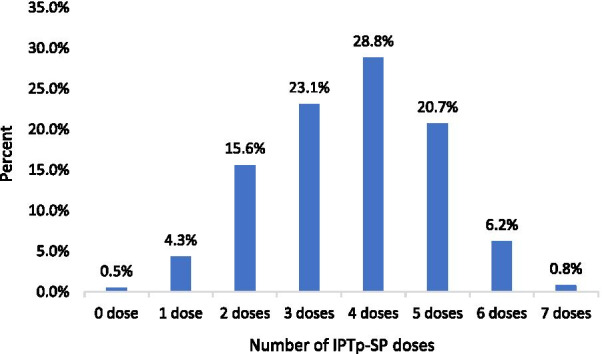
Distribution of IPTp-SP uptake levels against the drug’s dosage. IPTp-SP: Intermittent preventive treatment of malaria in pregnancy with sulphadoxine pyrimethamine

Overall, (370, 99.5%) of the respondents received at least one IPTp-SP dose. Of the 372 women, 79.6%, 95% *CI* 75.5%–83.7% received optimal IPTp-SP dose while (76, 20.4%) took sub-optimal IPTp-SP dose. The median IPTp-SP dosage was 4 (*IQR* = 2) doses. Most respondents (107, 28.8%) received four IPTp-SP doses (Fig. 1).

### Socio demographic, knowledge related and obstetric factors associated with IPTp-SP uptake

This study compared characteristics of women who received optimal IPTp-SP doses to those who received sub-optimal IPTp-SP. Table 3 shows the distribution of IPTp-SP uptake against the socio-demographic, obstetric and knowledge characteristics of women during pregnancy.

**Table 3:** Socio demographic, obstetric and knowledge characteristics associated with IPTp-SP uptake

Independent variables	All *N* = 372 *n* (%), Mean ± *SD*	Uptake of IPTp-SP		*P*-value
		Optimal *N* = 296 *n* (%), Mean ± *SD*	Sub-optimal *N* = 76 *n* (%), Mean ± *SD*	
Maternal age (years), mean(± *SD*)	26.3 ± 5.7	27.5 ± 5.8	25.3 ± 5.4	0.1
Age category				
15–24 years	160 (43.0)	121 (40.9)	39 (51.3)	0.29
25–34 years	173 (46.5)	142 (48.0)	31 (40.8)	
≥ 35 years	39 (10.5)	33 (11.1)	6 (7.9)	
Marital status				
Unmarried	87 (23.4)	62 (20.9)	25 (32.9)	0.04^a^
Married	285 (76.6)	234 (79.1)	51 (67.1)	
Woman's Education level				
Primary	142 (38.2)	108 (36.5)	34 (44.8)	0.48
Secondary	178 (47.8)	145 (49.0)	33 (43.4)	
Tertiary	52 (14.0)	43 (14.5)	9 (11.8)	
Woman's Employment status				
Not employed	252 (67.7)	193 (65.2)	59 (77.6)	0.17
Informal employment	95 (25.6)	82 (27.7)	13 (17.1)	
Formal employment	25 (6.7)	21 (7.1)	4 (5.3)	
Religion				
Catholic	20 (5.4)	13 (4.4)	7 (9.2)	0.15
Protestant	350 (94.1)	282 (95.3)	68 (89.5)	
Muslim	2 (0.5)	1 (0.3)	1 (1.3)	
Ward of Residence				
Busali	85 (22.9)	68 (23.0)	17 (22.4)	0.84
Wodanga	49 (13.2)	37 (12.5)	12 (15.8)	
North Maragoli	31 (8.3)	24 (8.1)	7 (9.2)	
Chavakali	64 (17.2)	52 (17.6)	12 (15.8)	
Sabatia West	60 (16.1)	46 (15.5)	14 (18.4)	
Lyaduywa Izava	83 (22.3)	69 (23.3)	14 (18.4)	
Residence				
Rural	287 (77.2)	224 (75.7)	63 (82.9)	0.16
Urban	85 (22.8)	72 (24.3)	13 (17.1)	
Gestation age at 1st ANC visit				
≤ 16 weeks	197 (53.0)	179 (60.5)	18 (23.7)	< 0.001^a^
> 16 weeks	175 (47.0)	117 (39.5)	58 (76.3)	
Frequency of ANC visits				
< 4 visits	84 (22.6)	33 (11.2)	51 (67.1)	< 0.001^a^
≥ 4 visits	288 (77.4)	263 (88.8)	25 (32.9)	
Parity				
1 child	142 (38.2)	108 (36.5)	34 (44.7)	0.41
2 children	82 (22.0)	66 (22.3)	16 (21.1)	
3+ children	148 (39.8)	122 (41.2)	26 (34.2)	
Experience of SP side effects				
No	197 (53.0)	154 (52.0)	43 (56.6)	0.29
Yes	173 (46.5)	142 (48.0)	31 (40.8)	
Not applicable	2 (0.5)	0	2 (2.6)	
Knowledge of MiP dangers				
Unknowledgeable	112 (30.1)	89 (30.1)	23 (30.3)	0.97
Low knowledge	136 (36.5)	110 (37.2)	26 (34.2)	
Moderate knowledge	94 (25.3)	73 (24.6)	21 (27.6)	
Adequate knowledge	30 (8.1)	24 (8.1)	6 (7.9)	
Knowledge of MiP prevention				
Unknowledgeable	5 (1.3)	4 (1.4)	1 (1.3)	0.86
Low knowledge	216 (58.1)	168 (56.7)	48 (63.2)	
Moderate knowledge	128 (34.4)	104 (35.1)	24 (31.6)	
Adequate knowledge	23 (6.2)	20 (6.8)	3 (3.9)	
Knowledge of IPTp-SP benefits				
Poor	77 (20.7)	50 (16.9)	27 (35.5)	< 0.001^a^
Good	295 (79.3)	246 (83.1)	49 (64.5)	
Knowledge of IPTp-SP start				
Poor	318 (85.5)	252 (85.1)	66 (86.8)	0.67
Good	54 (14.5)	44 (14.9)	10 (13.2)	
Knowledge of optimal SP dose				
Poor	183 (49.2)	135 (45.6)	48 (63.2)	0.01^a^
Good	189 (50.8)	161 (53.4)	28 (36.8)	

^a^Statistically significant result at 5% significance level. ANC: Antenatal care; IPTp-SP: Intermittent preventive treatment of malaria in pregnancy with sulphadoxine pyrimethamine; *SD*: Standard Deviation; SP: Sulphadoxine pyrimethamine

Pearson Chi-square and Fisher’s Exact text results show that IPTp-SP uptake was significantly associated with marital status (*P* = 0.04), gestation age at first ANC visit (*P* < 0.001), frequency of ANC visits (*P* < 0.001), knowledge of IPTp-SP benefits (*P* < 0.001) and knowledge of optimal SP dose (*P* = 0.01) (Table 3).

From bivariate logistic regression analysis, marital status (c*OR* = 1.8, 95% *CI* 1.0–3.1), gestation age at first ANC visit (c*OR* = 0.2, 95% *CI* 0.1–0.4), frequency of ANC visits (c*OR* = 17.1, 95% *CI* 9.4–31.2), knowledge of IPTp-SP benefits (c*OR* = 2.7, 95% *CI* 1.5–4.6) and knowledge of optimal SP dose (c*OR* = 2.0, 95% *CI* 1.2–3.3) were significant for optimal IPTp-SP uptake (Table 4). In multivariable logistic regression analysis, receipt of optimal IPTp-SP was higher among married women compared to unmarried women (a*OR* = 1.7, 95% *CI* 0.9–3.1), but insignificant (*P* = 0.09). Gestation age at ANC initiation and frequency of ANC visits remained significant factors when others were constant (*P* = 0.04), (*P* < 0.001) respectively. Women who started ANC attendance beyond 16 weeks gestation age were 60% less likely to receive optimal IPTp-SP (a*OR* = 0.4, 95% *CI* 0.2–0.9). Those with ≥ 4 ANC visits were about 17 times more likely to receive optimal IPTp-SP dose (a*OR* = 16.7, 95% *CI* 7.9–35.3). Maternal knowledge of IPTp-SP benefits (*P* < 0.001) and maternal knowledge of optimal SP dose (*P* = 0.03) remained significant predictors of IPTp-SP optimization after adjusting for other covariates. Women with good knowledge of IPTp-SP benefits had over two-fold increased likelihood to receive optimal doses than those with poor knowledge (a*OR* = 2.4, 95% *CI* 1.3–4.5). Those with good understanding of optimal SP dose were more likely to receive IPTp-SP3 + (a*OR* = 1.9, 95% *CI* 1.1–3.4) (Table 4).

**Table 4:** Socio-demographic, obstetric and knowledge predictors of uptake of SP during pregnancy

Predictors	Uptake of IPTp-SP		c*OR*, 95% *CI*	a*OR*, 95% *CI*	*P*-value
	Optimal *N* = 296	Sub-optimal *N* = 76			
	*n* (%)	*n* (%)			
Age category					
15–24 years	121 (40.9)	39 (51.3)	Reference		
25–34 years	142 (48.0)	31 (40.8)	1.4, 0.8–2.4		
≥ 35 years	33 (11.1)	6 (7.9)	1.8, 0.7–4.6		
Marital status					
Unmarried	62 (20.9)	25 (32.9)	Reference		
Married	234 (79.1)	51 (67.1)	1.8, 1.0–3.1	1.7, 0.9–3.1	0.09
Woman's Education level					
Primary	108 (36.5)	34 (44.8)	Reference		
Secondary	145 (49.0)	33 (43.4)	1.4, 0.8–2.4		
Tertiary	43 (14.5)	9 (11.8)	1.3, 0.6–2.9		
Woman's Employment status					
Not employed	193 (65.2)	59 (77.6)	Reference		
Informal employment	82 (27.7)	13 (17.1)	1.8, 0.9–3.4	1.7, 0.9–3.3	0.85
Formal employment	21 (7.1)	4 (5.3)	1.6, 0.5–4.9	1.4, 0.5–4.6	0.45
Religion					
Catholic	13 (4.4)	7 (9.2)	Reference		
Protestant	282 (95.3)	68 (89.5)	2.2, 0.8–5.7	2.5, 0.9–7.0	0.08
Muslim	1 (0.3)	1 (1.3)	0.5, 0.1–10.0	1.3, 0.1–25.3	0.87
Ward of Residence					
Busali	68 (23.0)	17 (22.4)	Reference		
Wodanga	37 (12.5)	12 (15.8)	0.8, 0.3–1.8		
North Maragoli	24 (8.1)	7 (9.2)	0.9, 0.3–2.3		
Chavakali	52 (17.6)	12 (15.8)	1.1, 0.5–2.5		
Sabatia West	46 (15.5)	14 (18.4)	0.8, 0.3–1.7		
Lyaduywa Izava	69 (23.3)	14 (18.4)	1.2, 0.6–2.7		
Residence					
Rural	224 (75.7)	63 (82.9)	Reference		
Urban	72 (24.3)	13 (17.1)	1.6, 0.8–3.1	1.60, 8–3.1	0.62
Gestation age at 1st ANC visit					
≤ 16 weeks	179 (60.5)	18 (23.7)	Reference		
> 16 weeks	117 (39.5)	58 (76.3)	0.2, 0.1–0.4	0.4, 0.2–0.9	0.04^a^
Frequency of ANC visits					
< 4 visits	33 (11.2)	51 (67.1)	Reference		
≥ 4 visits	263 (88.8)	25 (32.9)	17.1, 9.4–31.2	16.7, 7.9–35.3	< 0.001^a^
Parity					
1 child	108 (36.5)	34 (44.7)	Reference		
2 children	66 (22.3)	16 (21.1)	1.2, 0.6–2.3		
3+ children	122 (41.2)	26 (34.2)	1.5, 0.8–2.6		
Experience of SP side effects					
No	154 (52.0)	43 (56.6)	Reference		
Yes	142 (48.0)	31 (40.8)	1.3, 0.8–2.2		
Not applicable	0	2 (2.6)	N/A		
Knowledge of MiP dangers					
Unknowledgeable	89 (30.1)	23 (30.3)	Reference		
Low knowledge	110 (37.2)	26 (34.2)	1.0, 0.6–1.9	0.7, 0.4–1.5	0.38
Moderate knowledge	73 (24.6)	21 (27.6)	0.9, 0.5–1.8	0.6, 0.3–1.2	0.13
Adequate knowledge	24 (8.1)	6 (7.9)	1.0, 0.4–2.8	0.5, 0.2–1.6	0.28
Knowledge of MiP prevention					
Unknowledgeable	4 (1.4)	1 (1.3)	Reference		
Low knowledge	168 (56.7)	48 (63.2)	0.9, 0.1–8.0		
Moderate knowledge	104 (35.1)	24 (31.6)	1.1, 0.1–10.1		
Adequate knowledge	20 (6.8)	3 (3.9)	1.2, 0.1–13.7		
Knowledge of IPTp-SP benefits					
Poor	50 (16.9)	27 (35.5)	Reference		
Good	246 (83.1)	49 (64.5)	2.7, 1.5–4.6	2.4, 1.3–4.5	< 0.001^a^
Knowledge of IPTp-SP start					
Poor	252 (85.1)	66 (86.8)	Reference		
Good	44 (14.9)	10 (13.2)	1.2, 0.6–2.5		
Knowledge of optimal SP					
Poor	135 (45.6)	48 (63.2)	Reference		
Good	161 (53.4)	28 (36.8)	2.0, 1.2–3.3	1.9, 1.1–3.4	0.03^a^

^a^Statistically significant result at 5% significance level. ANC: Antenatal care; CI: Confidence interval; cOR: Crude odds ratio; IPTp-SP: Intermittent preventive treatment of malaria in pregnancy with sulphadoxine pyrimethamine; MiP: Malaria in pregnancy; SP: Sulphadoxine pyrimethamine

### Health service delivery factors influencing the uptake of optimal IPTp-SP

This study compared the health service characteristics with the outcome of IPTp-SP uptake.

From Table 5, higher proportion of women who received optimal IPTp-SP never missed being administered with SP during ANC visits compared to those who received sub-optimal SP doses (95.6% vs 86.8%, *P* = 0.01). There was no significant association between IPTp-SP uptake and water provision (*P* = 0.07), clean water (*P* = 0.38), clean cups (*P* = 0.40), enough cups (*P* = 0.46), ANC clinic working hours (*P* = 0.23), maternal fee (*P* = 0.08), waiting time (*P* = 0.34) and women’s relationship with ANC health workers (*P* = 0.41) (Table 5).

**Table 5:** Health service characteristics associated with uptake of IPTp-SP

Health service delivery variables	All *N* = 372 *n* (%)	Uptake of IPTp-SP		*P*-value
		Optimal *N* = 296 *n* (%)	Sub-optimal *N* = 76 *n* (%)	
Water provision at ANC clinic				
Never	4 (1.1)	4 (1.4)	0	0.07
Sometimes	3 (0.8)	3 (1.0)	0	
Always	352 (94.6)	282 (95.2)	70 (92.1)	
Not applicable	13 (3.5)	7 (2.4)	6 (7.9)	
Clean water at ANC clinic				
Never	1 (0.3)	1 (0.3)	0	0.38
Sometimes	2 (0.5)	2 (0.7)	0	
Always	352 (94.6)	282 (95.3)	70 (92.1)	
Not applicable	17 (4.6)	11 (3.7)	6 (7.9)	
Clean cups at ANC clinic				
Never	2 (0.5)	2 (0.7)	0	0.4
Sometimes	7 (1.9)	6 (2.0)	1 (1.3)	
Always	346 (93.0)	277 (93.6)	69 (90.8)	
Not applicable	17 (4.6)	11 (3.7)	6 (7.9)	
Enough cups at ANC clinic				
Never	44 (11.8)	37 (12.5)	7 (9.2)	0.46
Sometimes	13 (3.5)	11 (3.7)	2 (2.6)	
Always	298 (80.1)	237 (80.1)	61 (80.3)	
Not applicable	17 (4.6)	11 (3.7)	6 (7.9)	
ANC clinic working hours				
Half day	341 (91.7)	274 (92.6)	67 (88.2)	0.23
Full day	31 (8.3)	22 (7.4)	9 (11.8)	
SP administration at ANC clinic				
Never missed administering	349 (93.8)	283 (95.6)	66 (86.8)	0.01^a^
Ever missed administering	23 (6.2)	13 (4.4)	10 (13.2)	
Maternal service fee				
Never paid	363 (97.6)	291 (98.3)	72 (94.7)	0.08
Ever paid	9 (2.4)	5 (1.7)	4 (5.3)	
ANC clinic waiting time				
≤ 30 min	214 (57.5)	167 (56.4)	47 (61.8)	0.34
> 30 min	158 (42.5)	129 (43.6)	29 (38.2)	
Health worker-client relationship				
Poor	79 (21.2)	60 (20.3)	19 (25.0)	0.41
Good	293 (78.8)	236 (79.7)	57 (75.0)	

^a^Statistically significant result at 5% significance level. ANC: Antenatal care; IPTp-SP: Intermittent preventive treatment of malaria in pregnancy with sulphadoxine pyrimethamine

Bivariate analysis of health service predictors of optimal IPTp-SP uptake demonstrated that women who were always provided with water (c*OR* = 3.4, 95% *CI* 1.1–10.4), always provided with clean water (c*OR* = 2.2, 95% *CI* 0.8–6.0), sometimes found clean cups (c*OR* = 3.3, 95% *CI* 0.3–33.9), always found clean cups (c*OR* = 2.2, 95% *CI* 0.8–6.0), sometimes found enough cups (c*OR* = 1.2, 95% *CI* 0.2–6.6), always administered with SP (c*OR* = 3.2, 95% *CI* 1.4–7.7), queued for > 30 min (c*OR* = 1.3, 95% *CI* 0.8–2.2) and enjoyed good relationship with ANC health workers (c*OR* = 1.3, 95% *CI* 0.8–6.0) had increased odds of receiving optimal IPTp-SP (Table 6). However, at multivariable level, only women who never missed being issued with SP (a*OR* = 2.9, 95% *CI* 1.1–7.2) remained significantly associated with optimal IPTp-SP uptake.

**Table 6:** Health service predictors of uptake of IPTp-SP

Predictors	Uptake of IPTp-SP		c*OR*, 95% *CI*:	a*OR*, 95% *CI*:	*P*-value
	Optimal *N* = 296 *n* (%)	Sub-optimal *N* = 76 *n* (%)			
Water provision at ANC clinic					
Never	4 (1.4)	0	N/A		
Sometimes	3 (1.0)	0	N/A		
Always	282 (95.2)	70 (92.1)	3.4, 1.1–10.4	2.5, 0.8–8.4	0.13
Not applicable	7 (2.4)	6 (7.9)	Reference		
Clean water at ANC clinic					
Never	1 (0.3)	0	N/A		
Sometimes	2 (0.7)	0	N/A		
Always	282 (95.3)	70 (92.1)	2.2, 0.8–6.0		
Not applicable	11 (3.7)	6 (7.9)	Reference		
Clean cups at ANC clinic					
Never	2 (0.7)	0	N/A		
Sometimes	6 (2.0)	1 (1.3)	3.3, 0.3–33.9		
Always	277 (93.6)	69 (90.8)	2.2, 0.8–6.0		
Not applicable	11 (3.7)	6 (7.9)	Reference		
Enough cups at ANC clinic					
Never	37 (12.5)	7 (9.2)	Reference		
Sometimes	11 (3.7)	2 (2.6)	1.2, 0.2–6.6		
Always	237 (80.1)	61 (80.3)	0.9, 0.4–2.0		
Not applicable	11 (3.7)	6 (7.9)	0.4, 0.1–1.4		
ANC clinic working hours					
Half day	274 (92.6)	67 (88.2)	Reference		
Whole day	22 (7.4)	9 (11.8)	0.6, 0.3–1.4	0.6, 0.3–1.4	0.13
SP administration at ANC clinic					
Never missed administering	283 (95.6)	66 (86.8)	3.3, 1.4–7.7	2.9, 1.1–7.2	0.03^a^
Ever missed administering	13 (4.4)	10 (13.2)	Reference		
Maternal services fee					
Never paid	291 (98.3)	72 (94.7)	Reference		
Ever paid	5 (1.7)	4 (5.3)	0.3, 0.1–1.2		
ANC clinic waiting time					
≤ 30 min	167 (56.4)	47 (61.8)	Reference		
> 30 min	129 (43.6)	29 (38.2)	1.3, 0.8–2.2		
Health worker-client relationship					
Poor	60 (20.3)	19 (25.0)	Reference		
Good	236 (79.7)	57 (75.0)	1.3, 0.7–2.3		

^a^Statistically significant result at 5% significance level. ANC: Antenatal care; CI: Confidence interval; cOR: Crude odds ratio; IPTp-SP: Intermittent preventive treatment of malaria in pregnancy with sulphadoxine pyrimethamine; NA: Not Applicable; SP: Sulphadoxine pyrimethamine

## Discussion

### Optimal uptake of IPTp-SP

IPTp-SP optimal uptake in the study area approaches the national target of 80% though still far from universality. This high optimal IPTp-SP uptake is consistent with studies in Ghana and Sierra Leone [19, 20, 23]. However, studies in Uganda, Tanzania and Malawi that investigated IPTp-SP optimization found low uptake levels [17, 18, 30]. The high IPTp-SP uptake prevalence could be attributable to sustained efforts by the Kenyan government and development partners towards eliminating MiP and continued investment in maternal health. For instance, maternal health care services including IPTp-SP are free. There is continuous capacity building of health workers on MiP management and prevention. Also, MiP prevention messaging is being implemented. Optimal IPTp-SP uptake in this study is higher compared to 37.5% reported in the 2015 national malaria survey [21]. Scale up of MiP preventive strategies could account for this. Besides, this may indicate geographical variations in the intervention uptake across the country. Different study methodologies could also explain the difference. For example, the survey sampled respondents from the entire country while this study drew its sample from one Sub County.

### Socio demographic, knowledge related and obstetric determinants of optimal IPTp-SP uptake

In this study, late ANC initiators were less likely to receive optimal IPTp-SP. Similarly, in Tanzania first ANC booking before 17 weeks gestation age increased the odds of receiving IPTp-SP3+ [30]. Consistently, two studies in Zambia and Ghana reported ANC start date as a significant predictor of optimal IPT-SP uptake [24, 31]. However, a different study in Tanzania found no relationship between ANC initiation and IPTp-SP dosage [32] though it used a smaller sample size of 138. In line with other studies in Uganda, Tanzania, Malawi, Sierra Leone and Ghana, this study found that ≥ 4 ANC visits predicts optimal IPTp-SP uptake [17–20, 23, 24, 30]. The WHO calls for integration of IPTp-SP with initiatives for promoting focused ANC services [8]. Therefore, early ANC initiators are likely to achieve adequate visits, maximizing their contacts with health workers hence increased health education and SP administration. Evidently, pregnant women should be urged to initiate ANC visits earliest in the first trimester and adhere to all scheduled visits. Apparently, adequate ANC visits do not necessarily guarantee optimal IPTp-SP uptake. One-third of women with ≥ 4 visits in this study received sub-optimal IPTp-SP, possibly due to health service delivery deficiencies such as drug shortage.

IPTp-SP optimization could be realized when pregnant women are adequately and properly informed of the intervention. In Tanzania, Cameroon, Zambia and Ghana, maternal knowledge of IPTp-SP positively influenced the intervention’s maximum uptake [30, 31, 33, 34]. Similarly, this study found that women who understood IPTp-SP benefits and knew the recommended SP doses were more likely to receive optimal dosage. Good maternal knowledge of IPTp-SP empowers women to develop positive attitudes and perceptions towards adequate intervention uptake.

Marital status was significant before adjusting for other covariates. The findings from this study show as it does in Ghana that married women have higher odds of taking IPTp-SP3+ [35]. Though it did not consider IPTp-SP optimization, a different study in Kenya had similar findings [36]. Possibly, married women get financial and psychosocial support from their spouses towards ANC attendance. As evidenced by a study in Bungoma East district of Kenya, women who received support from their partners were more likely to receive > 1 SP dose [37]. It could also be that unmarried women get stigmatized for getting pregnant out of matrimony hence a need for their social protection.

Though no significant association, younger women, those with low education, the unemployed, rural residents and primiparous women had higher proportions of sub-optimal IPTp-SP uptake. As the unmarried, these are likely to be the 20.4% who received sub-optimal intervention. These women may lack adequate access to information and communication channels used for IPTp-SP promotion. Thus, mapping out these vulnerable women and using trained community health promoters to visit them, offer comprehensive MiP health education and encourage adequate ANC attendance is imperative. Information, education and communication materials developed in local dialect could buttress the above strategy. It may be desirable to facilitate peer education on MiP prevention among these vulnerable women. This study reinforces that women’s perceived benefits of IPTp-SP uptake outweigh their experience of the drug’s side effects. Thus, they should continue being educated that use of SP during pregnancy is safe and beneficial regardless of its side effects.

### Health service delivery predictors of optimal IPTp-SP uptake

Health service delivery dynamics are critical to appropriate IPTp-SP uptake [26]. Previous studies have argued that sub-optimal IPTp-SP dosage was mainly due to health system gaps [25–27]. In this study, SP administration at ANC clinic determined IPTp-SP3+ uptake. Health workers administer SP to eligible pregnant women and offer health education during ANC attendance. Ever missing to administer SP can create a sense of mistrust among women concerning the drug’s continued availability. Thus, subsequent ANC visits may be missed leading to sub-optimal IPTp-SP uptake. Besides, late ANC initiators who qualify for only three SP doses may receive sub-optimal dosage if they miss any dose. Inconsistent SP administration could be attributed to erratic drug supply. In Ghana, SP shortage was found to be a barrier to realizing high IPTp-SP uptake [25]. Also, health workers’ inadequacies to offer intensive IPTp-SP education due to high ANC clinic workload could contribute to the inconsistency.

## Study limitations

This study employed a cross sectional study design whose weakness is the impossibility to infer causation. Only women with live birth were included, missing an opportunity to study those with still birth. Responses were self-reported hence the possibility of recall bias. However, this was minimized by verification of some responses using ANC clinic attendance booklets. It was also minimized by excluding respondents with live birth beyond one year before the study. Health service delivery factors were not studied from health workers’ perspectives. Nonetheless, this study was household-based and used documented evidence to validate some variables. Household-based approach provided a chance to study participants who would have been missed had it been hospital-based due to poor care seeking behaviour among some women.

## Conclusions

This study has found high optimal IPTp-SP uptake in the study area. To sustain this, regular supportive supervision on adherence to IPTp-SP policies and guidelines is necessary. Gestational age at first ANC visit, frequency of ANC visits, maternal knowledge of IPTp-SP benefits, maternal knowledge of optimal SP dose and SP drug administration at ANC determines IPTp-SP3+ uptake. It is evident that efforts should be directed towards early and more frequent ANC visits. This study demonstrates that increasing awareness and knowledge of pregnant women on MiP preventive interventions through intensive, targeted health education and other behaviour change communication strategies is paramount to IPTp-SP optimization. Consequently, it is necessary to develop and implement a comprehensive MiP health education strategy including messages on early ANC attendance, adequate ANC visits, adherence to all ANC visits, MiP dangers, MiP prevention, IPTp-SP start date, IPTp-SP benefits and optimal IPTp-SP dosage. To strengthen service delivery, it is critical for health facilities to stock adequate SP drugs and health workers should consistently administer the drugs. Future studies considering larger samples and health workers’ perspectives of the health system delivery factors are recommended.

## Supplementary Information

**Additional file 1.** Data were collected using a semi-structured questionnaire. Data were collected on IPTp-SP uptake; women’s socio demographic, obstetric and knowledge related characteristics as well health service delivery factors.

**Additional file 2.** Data set used for the analysis and generation of the study results.

## Data Availability

All data generated and used for statistical analysis during this study are included in this article (Additional file 2).

## Abbreviations

ANC: Antenatal care
a*OR*: Adjusted odds ratio
*CI*: Confidence interval
c*OR*: Crude odds ratio
HIV: Human immunodeficiency virus
IPTp: Intermittent preventive treatment of malaria in pregnancy
IPTp-SP: Intermittent preventive treatment of malaria in pregnancy with sulphadoxine pyrimethamine
IPTp-SP3+: Optimal intermittent preventive treatment of malaria in pregnancy with sulphadoxine pyrimethamine
*IQR*: Interquartile range
LBW: Low birth weight
MiP: Malaria in pregnancy
N/A: Not applicable
*SD*: Standard deviation
SP: Sulphadoxine pyrimethamine
WHO: World Health Organization
