# Supplementary material for: Determinants of the uptake of intermittent preventive treatment of malaria in pregnancy with sulphadoxine pyrimethamine in Sabatia Sub County, Western Kenya
Source: Infect Dis Poverty. 2021 Aug 6;10:106. doi: 10.1186/s40249-021-00887-4 (PMC8343925; doi:10.1186/s40249-021-00887-4)
Supplement: Supplementary file 1 — Additional file 1. Data were collected using a semi-structured questionnaire. Data were collected on IPTp-SP uptake; women’s socio demographic, obstetric and knowledge related characteristics as well health service delivery factors. [file 40249_2021_887_MOESM1_ESM.docx]

**Determinants of the Uptake of Intermittent Preventive Treatment of Malaria in Pregnancy with Sulphadoxine Pyrimethamine in Sabatia Sub County, Western Kenya**

**Questionnaire Code: …………………………**

**Name of interviewer: ……………………………………………. Date: ………………………...**

**(Instruction: Tick ONLY ONE unless instructed otherwise)**

**Part I: Socio-Demographic Data**

1. How old are you? (age in years) ­­­­­­­­­­­­­­....................................
2. What is your current marital status?

□ Married

□ Living with partner

□ Single, never married

□ Separated

□ Widowed

1. What is the highest level of education have you attained?

□ None

□ Primary

□ Secondary

□ Tertiary

1. What is your religion?

□ Roman Catholic

□ Protestant/Other Christian

□ Muslim

□ No religion

Others (specify)……………………………………..................................

1. How many children have you given birth to alive?.........................................
2. What is your main occupation?

□ Formal employment

□ Informal employment

□ House wife

□ None

Others (specify)……………………………………..................................

1. Where do you live?

Ward …………………………………… Location ……………………………….

Sub location ……………………………. Village/Estate ………………………….

**Part II: IPTp-Sp Uptake**

1. How old was your last pregnancy when you first visited ANC clinic? (gestational age in weeks) ……………………………………….

(*Check and record the following from the Mother and Child Health Booklet*)

Date of LMP ………….. First date of ANC visit …………. Calculated no. of weeks …….

1. Were you given a drug like this (*show them SP tablets*) to swallow during any of the ANC visits?

□ Yes

□ No

1. If **yes** in Question 9 above, how many times during your entire pregnancy period were you given this drug? (*Confirm from the Mother and Child Health Booklet*) ……………………
2. For your answer in 10 above, where did you **always** swallow the drugs from?

□ Inside ANC clinic

□ Outside ANC clinic but within the health facility

□ At home

Others (specify)……………………………………………......................

1. If **inside the ANC clinic**, in Question 11 above, were you given water to take the drug with?

□ Yes always

□ Yes sometimes

□ Never

1. If **yes always/yes sometimes** in Question 12 above,
2. Was the water clean?

□ Yes always

□ Yes sometimes

□ Never

1. Were the cups clean?

□ Yes always

□ Yes sometimes

□ Never

1. Were the cups enough for each pregnant woman?

□ Yes always

□ Yes sometimes

□ Never

1. If **yes sometimes/never** in Question 12 above, how did you get the water you used to take the drug with?

□ Bought

□ Carried from home

□ Fetched from tap outside ANC clinic

Others (specify)……………………………………………………………

1. Did you ever experience any side effects of the drug after swallowing?

□ Yes

□ No

1. If **yes** in Question 15 above, which side effects did you experience? (***Tick ALL mentioned***)

□ Nausea

□ Vomiting

□ Headache

□ Dizziness

□ Loss of appetite

□ Stomach pain

□ Muscle weakness

Others (specify) ……………………………………………………

**Part III: Maternal Knowledge of Malaria in Pregnancy**

1. How is malaria brought about?

□ Mosquito bite

□ Being rained on

□ Eating newly harvested green maize

□ Chewing sugarcane

□ Eating raw mangoes

□ Witchcraft

Others (specify) ………………………………………………………

1. Is malaria a concern during pregnancy?

□ Yes

□ No

1. What can malaria cause to a pregnant woman and her child in the womb? (***Tick ALL mentioned***)

□ Anaemia

□ Preterm labour

□ Maternal death

□ Intrauterine death

□ Abortion/miscarriage

□ Premature birth

□ Low birth weight

□ Nothing

□ Do not know

Others (specify) ………………………………………………………

1. How can a pregnant woman prevent herself from getting malaria? (***Tick ALL mentioned***)

□ Sleeping under bed net

□ Using mosquito repellants

□ Taking fansidar/SP during ANC visit

□ Taking herbal medication

□ Wearing protective clothing especially at night

□ Do not know

Others (specify)……………………………………………………….

**Part IV: Maternal Knowledge of IPTp-SP**

1. Why are pregnant women given this drug (*show them SP tablets*) during ANC visits?

□ To prevent malaria

□ To increase the mother and baby’s weight

□ To increase mother and baby’s blood

□ Do not know

Others (specify)……………………………………………………….

1. What is the **best** gestational age (in weeks) for a pregnant woman to start taking this drug? (*show them the SP tablets*) ……………..
2. How many times during the entire period of pregnancy should a pregnant woman take this drug (*show them SP tablets*)? ………………………………………………
3. How did you know about the use of this drug (*show them SP tablets*) in pregnancy? (***Tick ALL mentioned***)

□ ANC staff/health worker

□ Radio

□ Television

□ Community Health Volunteer (CHV)

□ Spouse/partner

□ Fellow pregnant women

Others (specify)……………………………………………………….

**Part V: Health Service Delivery Factors**

1. Does your nearest health facility provide ANC clinic services?

□ Yes

□ No

1. What time are ANC clinic services usually accessible to pregnant women at the health facility/facilities where you attended ANC during your pregnancy?

□ Morning hours (8am to 1pm)

□ Afternoon hours (2pm to 5pm)

□ Morning to evening hours (8am to 5pm)

Others (specify) …………………………………………………………...

1. Did you ever attend ANC clinic but this drug (*show them SP tablets)* was **not** administered to you?

□ Yes

□ No

1. If **yes** in Question 27 above, what reason(s) were you told?

□ Drug out of stock

□ Not eligible for the medication

□ Reaction with the drug

□ None

Others (specify) …………………………………………………………..

1. Have you ever paid for any service during ANC visits?

□ Yes

□ No

1. If **yes** in Question 30 above,
2. How much did you pay on any single occasion? (in Ksh) ……………...
3. Who was paid?

□ ANC staff

□ Health facility cashier

□ Health facility in charge

□ Subordinate staff

Others (specify) …………………………………………………...

1. Was there a receipt for the payment?

□ Yes

□ No

1. How long did you spent on the queue during **most** of your ANC visits before being attended to by ANC staff? (time spent in minutes) ……………………
2. On **most** occasions at the ANC clinic;
3. Did the health worker greet you before attending to you?

□ Yes

□ No

1. Did the health worker spend enough time with you?

□ Yes

□ No

1. Did the health worker counsel or educate you on the benefits of taking this drug (show them the SP tablets)

□ Yes

□ No

1. Did the health worker give you a chance to ask him/her questions?

□ Yes

□ No

1. Were you comfortable asking the health worker questions?

□ Yes

□ No

1. Were your questions answered satisfactorily?

□ Yes

□ No

1. Did the health worker ever shout at you?

□ Yes

□ No

1. What do you suggest should be done to improve ANC and malaria control in pregnant women in this community?

………………………………………………………………………………………………………………………………………………………………………………………………………………………………………………………………………………………………………………………………………………………………………………………………………………………………………………………………………………………………………………………………………

**Thank You**
